# A Photolyase-Like Protein from *Agrobacterium tumefaciens* with an Iron-Sulfur Cluster

**DOI:** 10.1371/journal.pone.0026775

**Published:** 2011-10-31

**Authors:** Inga Oberpichler, Antonio J. Pierik, Janine Wesslowski, Richard Pokorny, Ran Rosen, Michal Vugman, Fan Zhang, Olivia Neubauer, Eliora Z. Ron, Alfred Batschauer, Tilman Lamparter

**Affiliations:** 1 Karlsruhe Institute of Technology (KIT), Botany I, Karlsruhe, Germany; 2 Philipps University, Institute of Cytobiology and Pathology, Core facility for Protein Spectroscopy, Marburg, Germany; 3 Philipps University, Plant Physiology and Photobiology, Marburg, Germany; 4 Agentek (1987) Ltd. Atidim Scientific Park, Tel Aviv, Israel; 5 Department of Molecular Microbiology and Biotechnology, The George S. Wise Faculty of Life Sciences, Tel Aviv University, Tel Aviv, Israel; 6 Humboldt University Berlin, Institute for Microbiology, Berlin, Germany; National Institute for Medical Research, United Kingdom

## Abstract

Photolyases and cryptochromes are evolutionarily related flavoproteins with distinct functions. While photolyases can repair UV-induced DNA lesions in a light-dependent manner, cryptochromes regulate growth, development and the circadian clock in plants and animals. Here we report about two photolyase-related proteins, named PhrA and PhrB, found in the phytopathogen *Agrobacterium tumefaciens*. PhrA belongs to the class III cyclobutane pyrimidine dimer (CPD) photolyases, the sister class of plant cryptochromes, while PhrB belongs to a new class represented in at least 350 bacterial organisms. Both proteins contain flavin adenine dinucleotide (FAD) as a primary catalytic cofactor, which is photoreduceable by blue light. Spectral analysis of PhrA confirmed the presence of 5,10-methenyltetrahydrofolate (MTHF) as antenna cofactor. PhrB comprises also an additional chromophore, absorbing in the short wavelength region but its spectrum is distinct from known antenna cofactors in other photolyases. Homology modeling suggests that PhrB contains an Fe-S cluster as cofactor which was confirmed by elemental analysis and EPR spectroscopy. According to protein sequence alignments the classical tryptophan photoreduction pathway is present in PhrA but absent in PhrB. Although PhrB is clearly distinguished from other photolyases including PhrA it is, like PhrA, required for *in vivo* photoreactivation. Moreover, PhrA can repair UV-induced DNA lesions *in vitro*. Thus, *A. tumefaciens* contains two photolyase homologs of which PhrB represents the first member of the cryptochrome/photolyase family (CPF) that contains an iron-sulfur cluster.

## Introduction

Photolyases are enzymes that repair UV-induced DNA lesions by using light energy. They can either repair CPDs, or pyrimidine-pyrimidone (6-4) photoproducts in single-stranded DNA (ssDNA) as well as in double-stranded DNA (dsDNA) [1]. Photolyases share high sequence similarity with cryptochromes (CRYs), which are blue/UV-A light photoreceptors that regulate growth, development and circadian rhythm in plants or act as transcriptional repressors of the circadian clock in mammals and some insects [2], [3].

Members of the CPF are widely distributed in all three kingdoms of life [1], [4]. According to their functions, they can be divided into three major classes: CPD photolyases, (6-4) photolyases and CRYs. Phylogenetically, the groups are further divided into the class I to class III CPD photolyases, plant CRYs, DASH CRYs, as wells as animal type I and II CRYs, respectively [4]–[7]. Animal CRYs are closely related to (6-4) photolyases, while plant CRYs form a sister group of the class III CPD photolyases. According to the present study, the CPF must be expanded by an additional class, which comprises, besides other proteins, PhrB from *A. tumefaciens*. We named this new class Fe-S bacterial cryptochromes and photolyases (FeS-BCPs). Interestingly, we identified FeS-BCP sequences in at least 350 bacterial organisms including many human and plant pathogens such as *Vibrio cholerae* and *Pseudomonas syringae*.

Photolyases generally bind two chromophores of which the catalytic FAD cofactor is common to all members [8]–[10]. The second chromophore in photolyases usually serves as photoantenna by absorbing light and transferring the excitation energy to the catalytic cofactor. So far, MTHF (pterin type), 8-hydroxy-7,8-didemethyl-5-deazariboflavin (8-HDF, deazaflavin type), FMN or FAD antennae have been found [1], [11], [12]. The FAD can be present either in the catalytic inactive, fully oxidized and semireduced radical states or in the catalytic active, fully reduced state. Reduction of the oxidized states to the fully reduced form *in vitro* is achieved by illumination of the enzyme in the presence of reducing agents. This process, known as photoactivation, involves the excitation of the flavin and subsequent electron hole hopping along the chain of three Trp residues (Trp triad) to the proteins surface, where the cationic or deprotonated neutral radical of surface Trp is reduced by a reductant in solution [13]. Whether photolyases need this type of photoactivation *in vivo* is a matter of debate [14]. The Trp triad is highly conserved in all CPF branches [15]–[19] but not in class II CPD photolyases where a tryptophan dyad was proposed to take over the same function [20]. In contrast, the FeS-BCPs seem to completely lack any Trp triad (this study).

We characterized two photolyase-related proteins PhrA and PhrB from *A. tumefaciens* regarding their spectral properties and demonstrate that PhrA repairs CPD lesions very efficiently *in vitro*, while both, PhrA and PhrB, seem to be required for *in vivo* photoreactivation. The main emphasis of this study was obtaining more information on the novel class of FeS-BCPs by phylogenetic analysis, structure prediction and spectroscopy, which led to the identification of an iron-sulfur cluster in PhrB.

## Results and Discussion

### *Agrobacterium tumefaciens* contains two photolyase-like proteins

By screening the *A. tumefaciens* genome for sequences with homology to photoreceptors we and others have already identified one cryptochrome/photolyase-related protein [5], [21], [22], which we named PhrA (gi: 17739623). An *A. tumefaciens* mutant in which *phrA* was knocked out has been used for physiological assays in an earlier study [21]. The second photolyase homolog has been found by transposon mutagenesis in a screen for light regulation of motility. Under the test condition, wild type cells showed a reduced motility in light as compared to darkness. Eight transposon mutants showed comparable motility in light and in darkness. The insertion loci of all mutants were sequenced and the results are summarized in Table 1. In the mutant *t1*, the *fliI* gene (gi: 17934468) is interrupted. This mutation resulted in a non-motile phenotype with no visible flagella, in accordance with an earlier study [23]. The mutants *t4* and *t7* displayed the same motility as the wild-type in darkness irrespective of the light conditions, while the motilities of mutants *t2*, *t3*, *t5*, *t6* and *t8* were lower compared to the wild type in darkness. In the *t5* mutant, the transposon was inserted into a gene annotated as photolyase-related protein. Sequence comparison showed that this protein is indeed homolog to known photolyases and CRYs although BLAST searches for *Escherichia coli* photolyase homologs failed to identify this protein in *A. tumefaciens*. We named the gene product PhrB (gi: 15158416). Results of the mutant studies suggested that PhrB might serve as a photoreceptor for light-regulated motility. The assumption that PhrB could act as photoreceptor is in line with recent finding on the regulation of photosynthesis gene transcription in *Rhodobacter sphaeroides* by CryB [24], which is a close homolog of *A. tumefaciens* PhrB.

**Table 1:** Transposon mutants of *A. tumefaciens*.

Gene/strain	GI	Insertion position	Ø L (mm)	Ø D (mm)	L/D	Definition
WT			3.5±0.1	6.8±0.1	0.5	
t1-*fliI*	17934468	560/1422	1.6±0.1	1.6±0.1	1.0	*fliI* flagellum-specific ATP synthase
t2-*wbiC*	15158598	32/921	5.6±0.3	5.4±0.1	1.0	*wbiC* putative glycosyl transferase
t3	15156942	2113/3384	5.7±0.2	6.0±0.3	1.0	putative transmembrane protein
t4-*secG*	15156708	10/489	6.6±0.3	7.2±0.1	0.9	*secG* preprotein translocase subunit
t5-*dprP*	15158416	1441/1524	4.6±0.3	4.6±0.2	1.0	*phrB*, formerly annotated as *dprP* deoxy-ribodipyrimidine photolyase related protein
t6-*fadL*	15156815	837/1290	4.7±0.3	4.7±0.2	1.0	*fadL* long-chain fatty acid transport protein
t7-*mcpA*	15157530	1114/2013	6.7±0.4	7.1±0.2	0.9	*mcpA* methyl-accepting chemotaxis protein
t8-*exoR*	17935609	120/795	5.6±1.5	4.6±0.5	1.2	*exoR* exopolysaccharide production negative regulator

Colony diameter (Ø) given in mm, n = 10±SE. (L) light, (D) dark incubated.

### PhrA is a member of the CPD III photolyases, PhrB belongs to a new class termed FeS-BCPs

**Figure 1:**
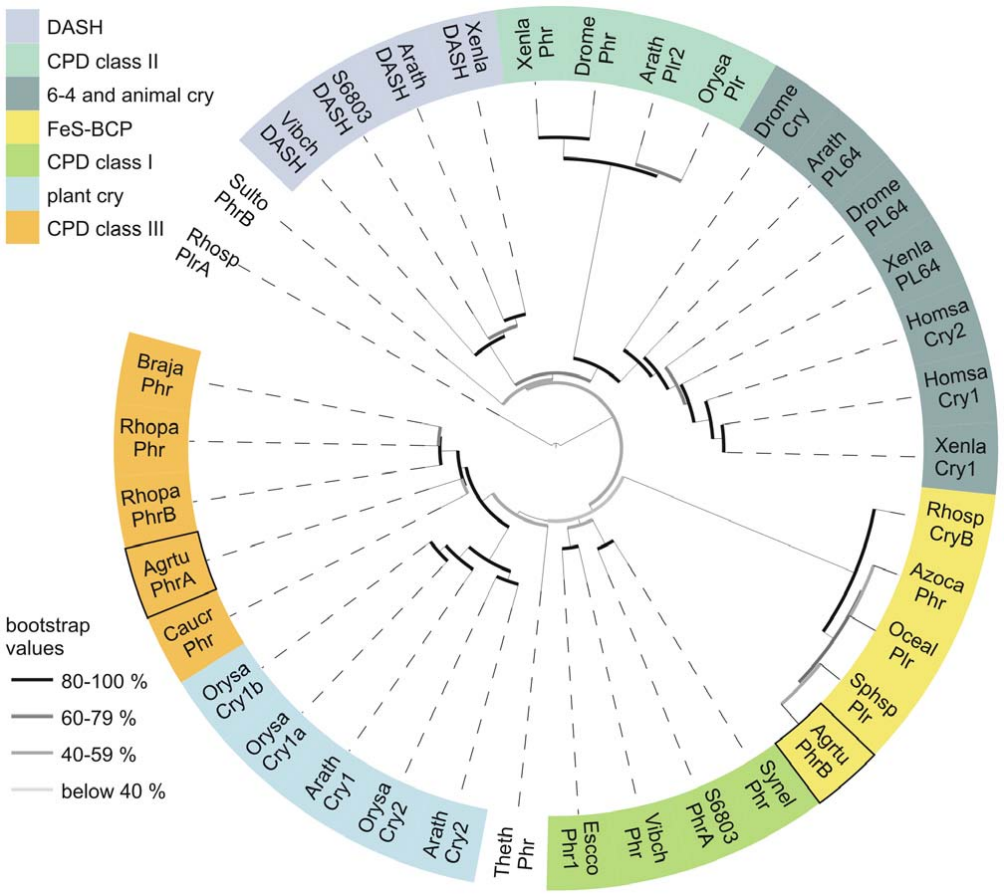
Phylogenetic tree of the cryptochrome/photolyase family. Protein sequences were aligned using ClustalX [61], 2.0.12 and the phylogenetic tree was constructed with PHYLIP. For details see materials and methods. Different bootstrap values are highlighted with different grey bars. The species names are abbreviated as follows: (*Arabidopsis thaliana* (Arath), *Agrobacterium tumefaciens* (Agrtu), *Azorhizobium caulinodans* ORS 571 (Azoca), *Bradyrhizobium japonicum* USDA 110 (Braja), *Caulobacter crescentus* (Caucr), *Drosophila melanogaster* (Drome), *Escherichia coli* (Escco), *Homo sapiens* (Homsa), *Oceanocaulix alexandrii* (Oceal), *Oryza sativa* (Orysa), *Rhodobacter sphaeroides* 2.4.1 (Rhosp), *Rhodopseudomonas palustris* (Rhopa), *Sphingomonas sp.* SKA58 (Sphsp), *Sulfolobus tokodaii* str. 7 (Sulto), *Synechococcus elongatus* PCC 6301 (Synel), *Synechocystis sp.* PCC 6803 (S6803), *Thermus thermophilus* HB8 (Theth), *Vibrio cholerae* (Vibch), *Xenopus laevis* (Xenla), Cry: cryptochrome, DASH: DASH cryptochrome, PL64: (6-4) photolyase, Phr: photolyase, Plr: photolyase-related protein, CPD: cyclobutan pyrimidine dimer, FeS-BCP: iron-sulfur cluster containing bacterial cryptochromes and photolyases.

We performed multiple sequence alignments (Fig. S1) and phylogenetic studies (Fig. 1). According to these studies, the CPF may be divided into seven classes: CPD photolyases class I to III, DASH CRYs, (6-4) photolyases together with animal CRYs, plant CRYs and a new class, named FeS-BCPs. PhrA is a close homolog of the lately characterized *Caulobacter crescentus* photolyase (Fig. 1), both belonging to the CPD class III photolyases, the sister group of plant CRYs [5].

**Figure 2:**
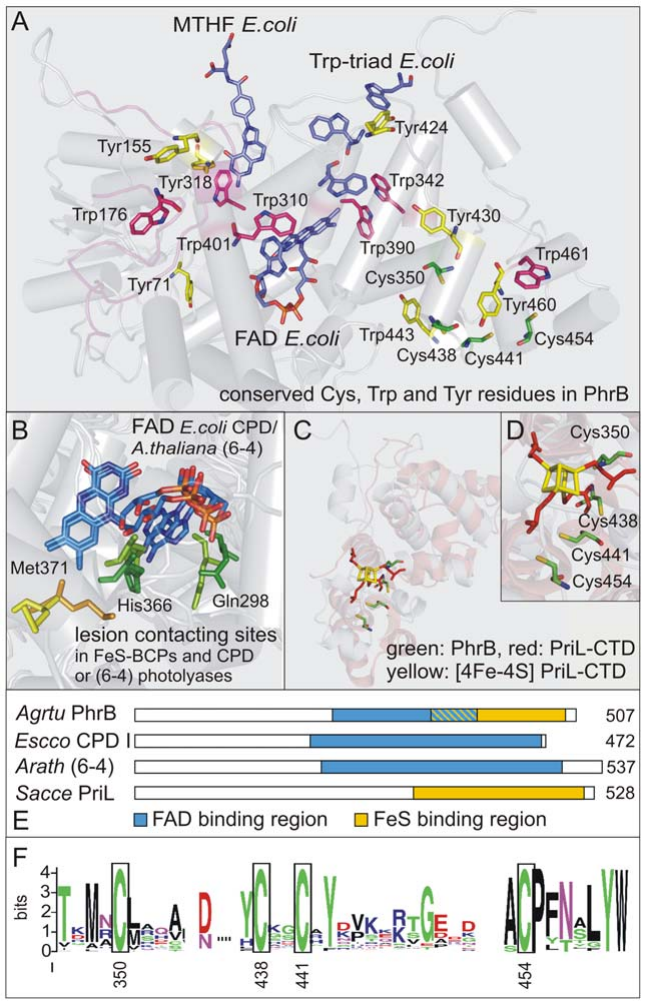
Three dimensional homology model of PhrB. The model was constructed with PS^2^ [25] using the *A. thaliana* Cry1 PHR crystal structure (PDB: 1U3D) as template. (A) Predicted overall structure of PhrB aligned to the *E. coli* CPD I photolyase (PDB entry 1DNP). Blue: *E. coli* chromophores (FAD, MTHF) and Trp-triad; red: conserved Trp residues in FeS-BCPs; yellow: conserved Tyr residues in FeS-BCPs; green: conserved Cys residues in FeS-BCPs. (B) Conserved lesion contacting sites of CPD or (6-4) photolyases and PhrB. Numbers display the residue position in *E. coli* CPD photolyase, *A. thaliana* (6-4) photolyase and *A. tumefaciens* PhrB. (C) Predicted PhrB structure (Cys350 to Asp507) aligned to the *S. cerevisiae* PriL CTD. (D) Close up of the (4Fe-4S) cluster (yellow) coordinated by four conserved Cys residues in PriL (red) and the conserved Cys residues in PhrB (green). (E) Schematic display of FAD (blue) and (FeS) cluster (yellow) binding region in the (6-4) photolyase from *A.thaliana* (*Arath* (6-4)), PhrB from *A. tumefaciens* (*Agrtu* PhrB), the CPD I photolyase from *E. coli* (*Escco* CPDI) and in the primase large subunit from *S. cerevisae* (*Sacce* PriL). Numbers display the protein length in amino acids. (F) Level of amino acid conservation in FeS-BCPs for the region of the (FeS) cluster coordination, constructed with WebLogo (http://weblogo.berkeley.edu/logo.cgi).

PhrB belongs to a yet undescribed phylogenetic class which is more distant to other proteins of the family (Fig. 1). Besides PhrB, this class comprises CryB from *R. sphaeroides* [24], as well as representatives in pathogens such as *V. cholerae* (gi: 15600828) or *P. syringae* (gi: 71735364), all together in 150 and 8 genera of eubacteria and euarchaea, respectively. To check for unique structural elements of FeS-BCPs, we performed a 3D homology modeling of PhrB using the prediction server PS^2^ [25] and the crystal structure of cry1 from *Arabidopsis thaliana* (PDB entry 1U3D) as a template. The model showed a very similar Cα-backbone fold to known crystal structures of photolyases and CRYs [19], [26]–[30]. However, the Trp residues of the classical electron transfer route (*e.g.* W306, W359 and W382 in *E. coli* photolyase; [16]) are missing in PhrB and its homologs (Fig. 2). An alternative tryptophan dyad shown for CPD class II photolyases (e.g. W360 and W381 in *Methanosarcina mazei* Mm0852; [20]), which further branches into at least two possible routes (i.e. either W388 or Y345 in *M. mazei* Mm0852), is also missing in PhrB. Alternatively, photoreduction of FAD with the participation of Tyr residues has been reported for some class I and (6-4) photolyases [31]. Altogether, six Trps and seven Tyrs are conserved in FeS-BCP proteins. These residues are highlighted in red (Trps) and yellow (Tyrs) in the 3D homology model of PhrB superimposed onto the *E. coli* photolyase structure (PDB entry 1DNP; Fig. 2A). However, there is no classical Trp-triad for the reduction of the FAD cofactor predicted by the PhrB homology model and may thus not exist in FeS-BCPs. An alternative pathway involving Trp and Tyr residues for the reduction of the FAD cofactor is also unlikely due to predicted distances larger than 10 Å between any Trp and Tyr residues.

As revealed by the superimposition of the PhrB homology model onto the structures of *E. coli* CPD I photolyase (PDB entry 1DNP) and *A. thaliana* (6-4) photolyase (PDB entry 3FY4), residues contacting the respective DNA lesion in PhrB are ambiguous (Fig. 2B). One of the two active-site His residues conserved in (6-4) photolyases is found also in PhrB (His366) and other FeS-BCPs (Fig. S1), whereas CPD I photolyases possess an Asn at this position [32], [33]. The other active-site His in (6-4) photolyases is replaced by a Met residue [34]. In PhrB, a Leu is found at the corresponding position but all FeS-BCPs have a conserved Met residue next to it (Met371 in PhrB, Fig. S1 and 2B). A Glu residue that is hydrogen-bonded to the 5′ Pyr of the lesion in CPD photolyases is replaced by a Gln306 in PhrB, which is also conserved in (6-4) photolyases [29], [34]. Thus, the specificity of FeS-BCPs for the respective lesion type (CPD or (6-4)) cannot be clearly drawn from the comparison above.

It was recently reported that the C-terminal domains of the large subunit of archaeal and eukaryotic primases (PriL-CTD) reveal a striking structural similarity to the active site region of photolyases and CRYs [35]. The region of similarity includes four cysteine residues necessary for the coordination of a (4Fe-4S) cluster in PriL-CTD [35]–[37]. PhrB also possess four clustered Cys residues at positions 350, 438, 441 and 454 (Fig. 2A and 2D) which are completely conserved in all FeS-BCPs (Fig. 2F). In FeS-BCPs, Cys_1_ is 86 to 89 amino acids away from Cys_2_, which is 2 amino acids in distance to Cys_3_. Between Cys_3_ and Cys_4_ 12 to 15 amino acids are located. This pattern for the conserved Cys residues resembles more that of archaeal - than that of eukaryotic primases. Moreover, a superimposition of the PhrB homology model onto the *Saccharomyces cerevisiae* PriL-CTD structure (PDB entry 3LGB) shows that Cys350 and Cys438 in PhrB occupy similar positions as Cys336 and Cys434 in the yeast PriL-CTD (Fig. 2C and 2D), although the positions and orientations of the other two Cys residues in the model does not exactly match with a cubiform geometry of a (4Fe-4S) clusters, as e.g. in the primase structure. Considering that the true tertiary structure is often slightly different from a homology model, the four conserved Cys residues in FeS-BCPs might be suitably positioned for the coordination of an iron-sulfur cluster. Moreover, besides the four conserved Cys residues, there is no other Cys in the C-terminal region of PhrB.

### Photoreduction of PhrA and PhrB

**Figure 3:**
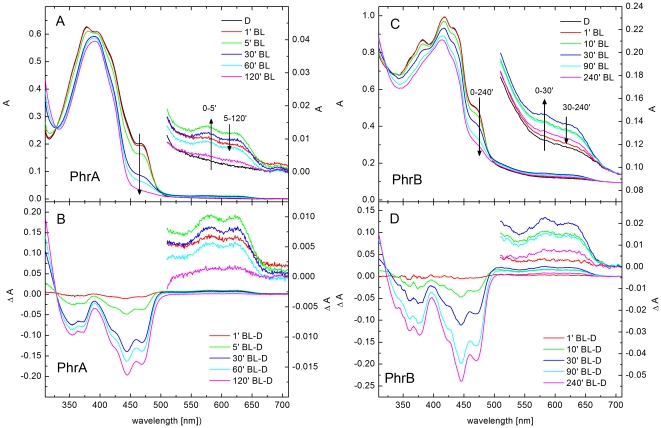
UV-vis spectroscopic characterization and photoreduction of *A. tumefaciens* PhrA and PhrB. (A, C) Initial spectra were measured for samples kept at 4°C in darkness for 24 h. Thereafter, spectra were taken at different time points after blue light (BL, 470 nm, 100 µmol m^−2^ s^−1^) irradiation from 1 min to 120 min for PhrA (A, B), and from 1 min to 180 min plus following 60 minutes of shorter wavelength blue light (405 nm, 150 *µ*mol m^−2^ s^−1^) for PhrB (C, D). The insets show an expanded scale of the absorption spectra in the 510 nm–710 nm range. (B, D) Light minus dark difference spectra calculated by subtracting the absorption spectra of the PhrA or PhrB sample kept in the dark from the absorption spectra of samples illuminated by BL at each time point.

Purified recombinant PhrA appears as a yellow chromoprotein. Figure 3A shows the absorption spectra of PhrA after incubation in darkness and at different time points after blue light illumination. The absorption spectrum of dark-adapted PhrA is characterized by a maximum at 380 nm, and shoulders at 400 and 470 nm. The latter shoulder is indicative for an oxidized FAD chromophore. Blue light irradiation in the presence of DTT results in an absorption decrease between 450 and 470 nm, attributed to the loss of oxidized FAD. The transient rise in absorption with maxima at 580 nm and 625 nm indicates the formation of the neutral radical form of FAD. Upon prolonged irradiation the absorption of the neutral radical decreases, indicative for the formation of the fully reduced FAD state. The light minus dark difference spectra (Fig. 3B) more clearly reveal these light-induced redox changes of the FAD cofactor without being masked by the strong absorption at 380 nm (Fig. 3A). This latter absorption peak in PhrA is attributed to the antenna cofactor which is most probably 5, 10-methenyltetrahydrofolate (MTHF; [38]). Reported MTHF absorption maxima in photolyases range from 377 nm for the *Saccharomyces cerevisiae* [39] to 390 nm for the *Neurospora crassa* enzyme [40]. 8-HDF can be excluded as its absorption maximum is at 440 nm [1]. Moreover, the recombinant enzymes were expressed in *E. coli*, which is not producing 8-HDF [1]. Based on the absorption values at 280 nm, 380 nm, and 470 nm and the known extinction coefficients of the apoenzyme, MTHF and oxidized FAD, a nearly stoichiometric ratio of both cofactors to the PhrA apoprotein was determined.

Heterologously expressed and purified PhrB has a brownish color. Figure 3C and 3D shows the absorption and difference spectra of PhrB kept in darkness and treated with blue light for different time periods. The absorption spectrum after dark incubation has maxima at 384 and 420 nm and shoulders at 435 and 470 nm. These absorption characteristics derive from two components: protein-bound FAD and a chromophore with rather broad bands which has significant absorption at 500–700 nm (see below). Blue light illumination resulted in an absorption decrease in the 345 nm to 500 nm range. Upon prolonged irradiation, a single broad absorption peak with a maximum at 415 nm remained (Fig. 3C). The difference spectra (Fig. 3D) show that the FAD cofactor in PhrB is photoreduced to the fully reduced FAD state as well.

The overall shape of the PhrB absorption spectrum differs from that of pterin-type photolyases or that of deazaflavin-type photolyases. For PhrB we found the highest spectral similarity with CryB from *R. sphaeroides* [24]. These authors showed that CryB comprises, in addition to FAD, another cofactor that is different from other flavins or MTHF but the cofactor remained unidentified. The absorption spectrum of PhrB also resembles that of the electron transfer flavoprotein-ubiquinone oxidoreductase [41]. This flavoprotein contains FAD and a (4Fe-4S) cluster as cofactors, which both contribute to the absorption in the spectral region of interest [41]. Proteins containing a (4Fe-4S) cluster and lacking flavin, such as bacterial ferredoxins, high-potential iron-sulfur proteins (HiPIPs) or the spore photoproduct lyase SplG, are characterized by a broad absorption extending up to 700 nm with one or several bands in the 300–500 nm range [42], [43]. A shift of the absorption maximum to a longer wavelength is seen for PhrB (415–420 nm) in comparison to PhrA (380 nm). The contribution of the extinction coefficient for PhrB at around 410 nm, determined by difference spectra, indicate 10,000 to 15,000 M^−1^ cm^−1^. Published extinction coefficients of (4Fe-4S) clusters at 410 nm vary between 11,900 M^−1^ cm^−1^ and 15,000 M^−1^ cm^−1^ [42]–[45]. Moreover, a significant absorption in the entire visible range was observed for PhrB under all conditions (Fig. 3C). These findings suggest that the second chromophore could be a (4Fe-4S) cluster.

### PhrB contains an (4Fe-4S) cluster

**Figure 4:**
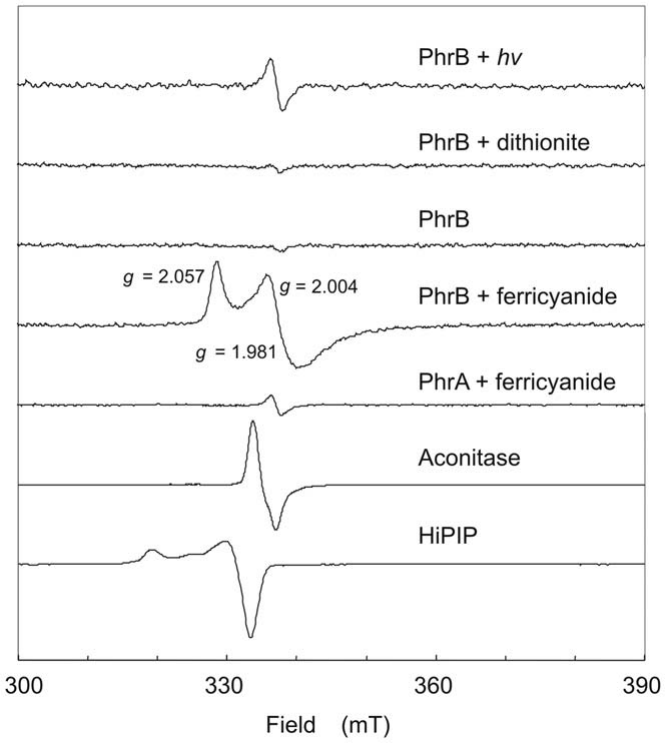
PhrB contains an Fe-S cluster. X band EPR spectra of PhrB and PhrA (normalized to equal protein concentration). Photoreduction was for 5 min under anaerobic conditions with white light (slide projector). Incubation with sodium dithionite (2 mM) or potassium ferricyanide (2 mM) was for 5 min at 23°C. For comparison the EPR spectra of (3Fe-4S)^1+^ in aconitase (H_2_O_2_-treated yeast mitochondria) and of the (4Fe-4S)^3+^ cluster in *Allochromatium vinosum* HiPIP are shown (arbitrary scaling). EPR conditions: microwave power, 0.2 mW; microwave frequency 9.458 GHz; modulation frequency, 100 kHz; modulation amplitude, 1.25 mT; temperature, 15 K.

Chemical analysis on as-isolated PhrB gave an iron and sulfide content of 3.9±0.4 atoms iron and 2.7±0.2 moles sulfide per mol protein. The latter value might be underestimated since protein molecules with broken-down clusters retain Fe but can inadvertently loose H_2_S. Since the superposition of other cofactors in the visible spectral range complicated direct detection of the Fe-S cluster, we employed EPR spectroscopy. Except for a weak organic radical signal, PhrB did not exhibit EPR signals in the dithionite-treated, anaerobically photoreduced and as isolated form, but upon brief treatment with potassium ferricyanide a strong signal with g-values of 2.057, 2.004 and 1.981 developed (Fig. 4). The signal was detected between 4 and 30 K, but broadened beyond detection at higher temperature. PhrA lacked EPR signals with the exception of a weak flavin radical signal. EPR linewidths, *g*-values and microwave power of half-saturation (15 mW at 10 K) of PhrB are unlike the parameters for radicals, but are typical for EPR signals of spin-coupled paramagnetic ions in Fe-S clusters. Double integration versus a Cu^2+^ standard gave 0.6 spin-coupled centers per PhrB, so the signal cannot derive from a contaminant but is from a moiety present in almost stoichiometric proportion. The likely source of the signal is a Fe-S cluster composed of approximately 4 Fe and 3 S^2−^ ions, based on the chemical analysis. Occurrence of an EPR signal in the oxidized form excludes (2Fe-2S)^1+/2+^ and (4Fe-4S)^1+/2+^ clusters as in plant or bacterial ferredoxins. This leaves two possibilities: (3Fe-4S)^0/1+^ or (4Fe-4S)^2+/3+^ clusters occurring in aconitase and high potential iron-sulfur proteins (HiPIP), respectively. These Fe-S clusters have an EPR signal upon oxidation (Fig. 4, lowest traces) with EPR spectral properties similar with, but not identical to oxidized PhrB. The situation is reminiscent of human and yeast primases [46], [47] where crystal structure and elemental analysis is in agreement with a (4Fe-4S)^2+^ cluster in the native state, but EPR signals upon oxidation are neither like regular (3Fe-4S)^1+^ clusters in aconitase nor like (4Fe-4S)^3+^ clusters in HiPIP. For *Bacillus subtilis* AddAB helicase/nuclease [48], which contains a (3Fe-4S) or (4Fe-4S) cluster, similar EPR spectroscopic observations were made. In summary, we propose that PhrB contains a (4Fe-4S)^2+^ cluster which upon oxidation is converted to EPR active species.

This is the first experimental evidence that a member of the CPF contains an iron-sulfur cluster. Iron-sulfur clusters are well known for their role in electron transfer reactions of photosynthesis and respiration. Furthermore several DNA repair enzymes like MutY [49], endonuclease III [50], DNA helicases [51] and spore photoproduct lyases [43], [44] contain iron-sulfur clusters. MutY and endonuclease III are involved in base excision repair and are proposed to use their (4Fe-4S) clusters for efficient lesion detection [52]. DNA repair helicases, such as XPD and FancJ, belong to the group of nucleotide excision repair enzymes. The iron-sulfur clusters in both are found within a conserved domain near the N-terminus that serves to separate DNA strands as the protein translocates along the DNA [51]. Spore photoproduct lyases repair a special type of lesions, so-called spore photoproducts [53], [54] which are unique to spore-forming microorganisms [55], [56]. Buis *et al.* [44] suggested that the repair mechanism in this case involves a reduced (4Fe-4S)^1+^ cluster and *S*-adenosylmethionine as catalytic cofactor, with a mechanism in which the 5′-deoxyadenosyl radical is reversibly generated. The (4Fe-4S) cluster in PhrB may be involved in electron transfer to the FAD cofactor or alternatively may play a role in the recognition process similar to other (4Fe-4S) cluster enzymes involved in DNA repair.

### *In vivo* photoreactivation in *Agrobacterium tumefaciens* depends on both, PhrA and PhrB

**Figure 5:**
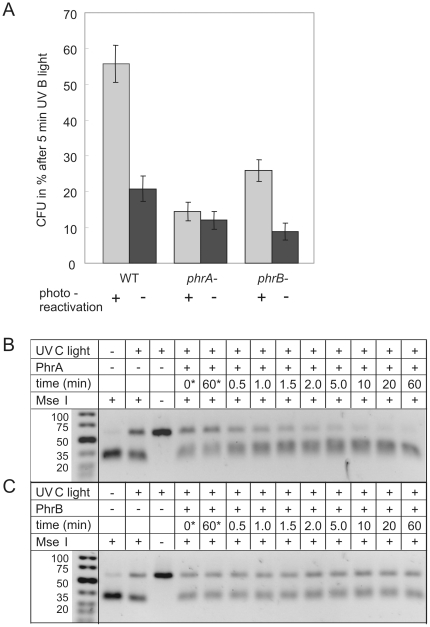
Photorepair of UV-lesions. (A) *In vivo* photoreactivation test. *A. tumefaciens* cells of the wild type (WT), the *phrA^−^* and the *phrB^−^* mutant were spread on LB agar plates, irradiated with UV-B light for 5 min and immediately transferred to darkness (−) or subjected to photoreactivating white light (+). Mean values of CFU ± SE; n = 8 to 14. (B and C) *In vitro* repair of pyrimidine dimers by PhrA and PhrB. Thymine dimers were created by UV-C illumination of the DNA substrate (first row, +/− UV-C), mixed with enzyme (second row) and incubated for the indicated times in photoreactivating blue light (405 nm, 150 *µ*mol m^−2^ s^−1^) or in darkness (*) (third row). Photorepaired DNA can be digested by *Mse*I (fourth row, +/− *Mse*I). Shown are representative gels from three independent repair experiments using PhrA or PhrB.

To test for photolyase activity of PhrA and PhrB, we performed *in vivo* tests with wild type *A. tumefaciens* cells and the *phrA^−^* and *phrB^−^* mutants (Fig. 5A). As in the classical photoreactivation tests, cells were treated with UV-B irradiation and subsequently placed in darkness or photoreactivating white light. The effect of photorepair was best seen after 5 min UV-B treatment, where 21±4% cells survived in darkness and 56±5% survived after photoreactivation (Fig. 5A). The survival rate of the *phrA^−^* mutant was, irrespective of photoreactivating light, lower than that of the wild type kept in darkness after UV-B treatment. Thus, lack of PhrA cannot be compensated by PhrB. This may be indicative for a weak CPD-repair activity of PhrB or for the repair of (6-4) photoproducts by PhrB. Such (6-4) lesions constitute only 10–20% of the total photoproducts, compared to CPDs, which make up 80–90% of the photolesions [1]. Alternatively, the PhrA activity or expression might be regulated by PhrB. The *phrB^−^* mutant showed a strongly attenuated photoreactivation (26±3% survival), much less efficient than that of the wild type (56±5% survival). The survival rate of the photoreactivated *phrB^−^* mutant was 17% above the *phrB^−^* dark control. Thus, a photorepair activity, most likely mediated by PhrA, can be still detected in this mutant. These results indicate that both PhrA and PhrB are involved in photoreactivation. For CryB, the close *R. sphaeroides* homolog of PhrB, *in vivo* photoreactivation tests with the knock out mutant revealed also a reduced photoreactivation in comparison to the wild type [57].

### PhrA repairs *in vitro* UV-C damaged dsDNA

To test whether PhrA and PhrB are capable of binding and repairing CPD lesions in dsDNA, we performed *in vitro* studies with recombinant proteins and UV-C treated dsDNA probes containing such lesions (Fig. 5B and 5C). It should be noted that the probe used here comprises 15 sites with at least two neighboring pyrimidines and is therefore heterogeneous in regard to the number, type and position of CPDs formed by UV-C. However, each strand contains only one T<>T dimer within the *Mse*I recognition sequence. Thus, a restriction site restoration assay can be used to monitor the photorepair process, although the actual number of repaired lesions can be substantially higher compared to the number of restored *Mse*I sites. The reaction mixtures of experiments shown in Fig. 5B and 5C contained 400 nM DNA and 17 nM PhrA or PhrB, respectively. Within one hour of blue light treatment at 4°C, PhrA repaired all damaged *Mse*I sites whereas without photoreactivating light no repair was detected (Fig. 5B). Furthermore, we tested the repair of CPDs and T(6-4)T photoproducts in a UV-treated single-stranded oligo(dT)_18_ spectrophotometrically at 265 nm and 325 nm. In such an assay photoreduced PhrA was found to repair up to 75% of CPDs in ssDNA within one hour but no T(6-4)T photoproducts, detected as an absorption increase at 265 nm (data not shown). Hence, PhrA fulfils all the characteristics of a *bona-fide* CPD photolyase – it can bind to and repair CPDs in ssDNA as well as in dsDNA. When PhrB was used in the restriction site restoration assay, damaged DNA was apparently not repaired (Fig. 5C). The same result was found for a very high PhrB to probe ratio of 20∶1. Moreover, the repair of CPDs or T(6-4)T photoproducts in single-stranded oligo(dT)_18_ by photoreduced PhrB was tested. Again, no clear repair of UV-damaged DNA was detected (data not shown). It is possible that a hitherto unidentified accessory protein is required, which enhances binding and/or catalytic activity of PhrB. Thus, under the tested conditions no *in vitro* repair of these lesions in ssDNA and dsDNA by PhrB was observed.

### Conclusions

*A. tumefaciens* contains two different types of photolyase homologs which are both required for photorepair activity *in vivo*. These two proteins are found at evolutionary exposed positions: While PhrA belongs to a sister group of plant CRYs and its further analyses could contribute to our understanding of plant CRY evolution, PhrB belongs to a newly characterized group of the CPF that contains an iron-sulfur cluster besides the conserved FAD as cofactors. We eagerly await structural characterization for experimental distances between the cofactors. From thissuch results a more precise conclusion on the function or a discrimination between electron transfer and DNA recognition function can be drawn.

## Materials and Methods

### Bacterial strains and growth conditions

Bacterial strains used in this study were *A. tumefaciens* C58 harboring the Ti nopaline plasmid (DSMZ - the German Resource Centre for Biological Material, http://www.dsmz.de/), its knockout mutants *phrA* ^−^ [21] and *phrB* ^−^ (this study) as well as following *E. coli* strains: XL1 blue (Stratagene), ER2566 (NEB) for recombinant expression and BW20767 [58] for transposon mutagenesis. Bacteria were grown at 14–37°C (*E. coli*) and 28°C (*A. tumefaciens*) in Luria-Bertani (LB) medium. Depending on the resistance, 50 µg/ml kanamycin (Kan), 250 µg/ml spectinomycin (Spc), 100 µg/ml rifampicin (Rif) or 100 µg/ml ampicillin (Amp) were added.

### Transposon mutagenesis, mutant screen and transposon insertion cloning

Transposon mutagenesis was performed as previously described [59]. Briefly, plasmids were transferred into *A. tumefaciens* recipients by conjugation from *E. coli* BW20767 carrying the plasmid pRL27. To this end, donor and recipient strains were grown to an OD_600_ of around 0.8, mixed and collected by filtration using a 0.45 µm analytical filter (Nalgene). This filter was incubated overnight at 28°C on LB agar medium containing Kan. After incubation, the cells were resuspended in liquid LB medium and plated onto LB medium containing Kan and Rif for selection of *A. tumefaciens* cells that gained the transposon plasmid pRL27 by conjugation.

The screen for *A. tumefaciens* mutants with altered motility was performed on soft agar plates. Bacteria were grown on solid media containing Kan, transferred with a tooth pick onto LB plates containing 0.3% agar and incubated for 24 hours in white light (Osram L 36 W/765 cool daylight, 150 *µ*mol m^−2^ s^−1^) or in darkness.

One step cloning of Tn*5*-RL27 insertions was performed as described in Larsen *et al.* [59]. Genomic DNA from a transposon-induced mutant was digested with *Bam*HI and subsequently treated with T4 DNA ligase for introduction into *E. coli* DH5α/λ*pir*. Transposon junction plasmids were isolated from selected transformants and subjected to sequencing using the transposon specific primers tpnRL17–1 and tpnRL13–2 (Table S1). There have been no new data created for deposition in the GenBank. Sequences were then compared to the protein sequence database (GenBank) using the BlastX algorithm [60] for identification of the mutated gene (Table 1). The Tn*5*-RL27 insertions were confirmed by Southern blot analysis using the digoxygenin labelling system (Roche).

### Sequence analyses

All sequences of photolyase-related proteins were retrieved from public databases *via* the National Center for Biotechnology Information web site (www.ncbi.nlm.nih.gov). Multiple sequence alignments were carried out using ClustalX [61], version 2.0.12 and ClustalW2 (http://www.ebi.ac.uk/Tools/clustalw2/index.html). For the construction of the phylogenetic tree, a ClustalX alignment was performed with 37 selected prototypical sequences. In the “multiple alignment parameters”, the “gap opening” was set to 50 and the “BLOSUM series” was selected as weight matrix. The phylogenetic tree was constructed with the PHYLIP program package version 3.6 (Z) using the SEQBOOT, PROTDIST, FITCH and CONSENSE utilities with default parameters and drawn with iTOL [62]. The number of bootstrapping datasets was set to 100. The abbreviations of species names are given in the legend of Fig. 1.

### Recombinant expression of PhrA and PhrB

For the cloning of a PhrA expression vector, the gene *phrA* (gi: 15156266, accession: AAK87020) was PCR-amplified with Taq polymerase (Sigma), using the primers *phrA* 5′, *phrA* 3′ (Table S1) and genomic *A. tumefaciens* DNA as template. Purified PCR products were cloned into the linear pQE30UA expression vector (Qiagen) and transformed into *E. coli* XL1 blue cells. For cloning of an expression vector for PhrB, the gene *phrB* (gi: 15158416; accession: AAK88685) was PCR-amplified with Phusion polymerase (NEB) using genomic DNA as template and the primers *phrB* NdeI 5′ and *phrB* NotI 3′ (Table S1). The NdeI/NotI digested and purified PCR product was ligated into the corresponding restriction sites of vector pET-21b (Novagen/Merck) and transformed into *E. coli* ER2566 cells. The expression vectors encode for a protein with an N-terminal His_6_-tag (*phrA*) or a C-terminal 6× His_6_-tag (*phrB*). Correct cloning was confirmed by DNA sequencing. There have been no new data created for deposition in the GenBank.

For protein expression, bacteria were grown in 3 litres LB with Amp at 37°C to an OD_600_ of 0.6 to 0.8. Thereafter, the cells were shaken at 14°C (PhrA) or 28°C (PhrB). Expression was induced by the addition of isopropyl β-D- thiogalactopyranoside (IPTG) to a final concentration of 100 µm. After one (PhrB) or three days (PhrA) of expression, cells were collected by centrifugation and washed with extraction buffer (50 mM Tris/HCl, 5 mM EDTA, 300 mM NaCl, 10% w/v glycerol, pH 7.8), centrifuged again and resuspended in the same buffer. Cells were disrupted using a French pressure cell 1300 bar and the cell debris was pelleted at 4°C and 25,000 *g* for 10 min. Ammonium sulphate precipitation was performed by adding 93% (final volume) of AmS buffer (3.3 M ammonium sulphate, 50 mM Tris/HCl, pH 7.8) to the protein solution. Precipitated proteins were pelleted at 25,000 *g* for 30 min, resuspended in the wash buffer (WB: 50 mM Tris/HCl, 10 mM imidazole, 300 mM NaCl, 10% w/v glycerol, pH 7.8) and centrifuged again. Supernatant was applied to a WB-equilibrated column packed with Ni^2+^-NTA agarose matrix (Qiagen). After washing the column with WB, bound protein was eluted with elution buffer (EB: 50 mM Tris/HCl, 250 mM imidazole, 300 mM NaCl, 10% w/v glycerol, pH 7.8). The eluate was concentrated by ammonium sulphate precipitation and the protein was resuspended in extraction buffer. PhrB was then subjected to size-exclusion chromatography using a 200 ml Superdex 200 HR 10/30 column (GE Healthcare) and the extraction buffer for separation. PhrB containing fractions were again subjected to an ammonium sulphate precipitation and resuspended in the extraction buffer.

### Characterization of PhrA and PhrB by UV-vis spectroscopy

Spectrophotometric measurements of recombinant proteins were performed in a Jasco V550 photometer at 4°C. PhrA or PhrB were incubated for 24 h in darkness at 4°C in order to get the fully oxidized FAD cofactor. Thereafter, 10 mM and 20 mM DTT (final concentration) was added to PhrA and PhrB solutions, respectively. For photoreduction, we used either a light emitting diode with a maximum emission wavelength of 470 nm (HLMP-HB57-LMC, Avago Technologies) and a light intensity of 55 *µ*mol m^−2^ s^−1^ or a light emitting diode with an emission maximum at 405 nm (GB333UV1C/L1, Farnell Diodes) and a light intensity of 100 *µ*mol m^−2^ s^−1^. Spectra were taken at different time points ranging from 1 min to120 min of 470 nm illumination for PhrA and from 1 min to 180 min of 470 nm illumination followed by additional 0–60 min of 405 nm illumination for PhrB. The difference spectra were calculated by subtracting the absorption spectra of the dark-incubated proteins from the absorption spectra after blue light illumination. For the calculation of the cofactor to protein ratios, absorption values at 280 nm, 380 nm, and 470 nm and the known extinction coefficients of the apoenzyme, MTHF, and oxidized FAD (FAD_ox_) (ε_Apo 280 nm_ 122,310 M^−1^ cm^−1^, ε_FADox 280 nm_ 17,638 M^−1^ cm^−1^, ε_FADox 380 nm_ 8917 M^−1^ cm^−1^, ε_FADox 470 nm_ 9429 M^−1^ cm^−1^, ε_MTHF 280 nm_ 9895 M^−1^ cm^−1^, ε_MTHF 380 nm_ 23050 M^−1^ cm^−1^) were used.

### Determination of iron and acid-labile sulphide in PhrB

To estimate the concentration of iron in protein solutions, a protocol modified from Rad *et al.* [63] was used. A 100 µl protein solution (35 µM) dissolved in the extraction buffer was mixed with 5 µl concentrated HCl and heated for 10 min to 80°C to release the cofactors. Control reactions contained either PhrA or FAD at the same concentrations or buffer only. After 10 min of centrifugation at 13,000 *g*, 100 µl of the supernatant were mixed with 1.3 ml of 500 mM Tris/HCl, pH 8.5, 100 µl 5% ascorbic acid and 400 µl of 0.1% bathophenanthroline disulphonic acid was added. The reactions were incubated for 1 h at room temperature and centrifuged again (10 min at 13,000 *g*). The absorption of the reaction mixture was measured in the 250–650 nm range. A standard curve was constructed using the Fe^2+^ solutions (ammonium ferrous sulphate hexahydrate) in the range of 0–500 µM.

### EPR spectroscopy

PhrA (28 µM) and PhrB (13 µM) in extraction buffer were shock-frozen in quartz tubes with liquid nitrogen. EPR spectra were recorded with a Bruker EMX-6/1 EPR spectrometer with ER-041 XG X band microwave bridge, standard universal TE102 rectangular cavity, ER-041-1161 microwave frequency counter, ER-070 6-inch magnet and EMX-032T Hall probe. Samples were kept at low temperature with an ER-4112HV Oxford Instruments variable temperature helium flow cryostat. The WINEPR software supplied by the manufacturer was used for data collection, subtraction of baselines and conversion into ASCII format.

### *In vivo* photorepair tests

To test for *in vivo* photorepair, *A. tumefaciens* cells of the wild type, the *phrA^−^* and *phrB^−^* mutant were grown in LB medium at 28°C to an OD_600 nm_ of 0.3. The cell suspensions were diluted 1∶100,000 and 50 µl were spread on LB agar plates. The plates were irradiated with UV-B light (Philips TL40/W12; 50 cm distance) for the indicated times. Half of the plates were immediately transferred to darkness, the other half was subjected to photoreactivating white light (80–100 µmol m^−2^ s^−1^) for 30 minutes. The plates were incubated for two days in darkness at 28°C and the number of colonies was determined.

### *In vitro* photolyase activity

Synthesis of the dsDNA probe was performed by PCR using the partially overlapping primers probe 5′, probe 3′ (Table S1) and Taq polymerase (NEB) (no extra template). The predicted sequence of the PCR product is: 5′ATAGGACGATGCTGGATGTCGAGGTGTCGTTAATGTGGAGCTGTAGGTCGTACTATACGG 3′. It contains a single *MseI* site (underlined) overstretching a T-T dimer but represents a heterogeneous mixture of duplexes in regard to the number, type and position of CPDs. The PCR products were supplemented with 20% acetone and degassed for 15 min with argon. CPDs were created by illuminating the probe for 4 h with UV-C on ice (UV-C: GE Healthcare, G15T87B, 15 W; 10 cm distance). Acetone was then removed by heating for 10 min to 65°C. Proteins had been photoreduced immediately before the repair assay by blue light to achieve their reduced flavin state. The repair assay was performed in following repair buffer: 50 mM Tris/Cl, 100 mM NaCl, 1 mM EDTA, 10 mM DTT, 100 µg ml^−1^ BSA, 10% (w/v) glycerol pH 7.6 at 4°C. After an incubation of 20 min in the dark on ice, each reaction mixture was divided into 2 parts. One part was illuminated with 150 *µ*mol m^−2^ s^−1^ of 405 nm blue light (GB333UV1C/L1, Farnell Diodes), the other part was kept in the dark. Aliquots were taken at the indicated time points. Subsequently, proteins were inactivated by heating to 95°C for 10 min and the DNA probe was digested with *Mse*I (NEB). DNA with a T<>T dimer in the *Mse*I site cannot be cleaved by the restriction enzyme in contrast to the photorepaired probe. After electrophoresis on 4% TBE agarose gels (NuSieve, Lonza), DNA bands were visualized by SYBR Safe fluorescence (Invitrogen). Several control reactions, such as digested undamaged DNA as well as digested and undigested damaged DNA, were included in each set of experiment. The repair of thymine dimers in single-stranded DNA was monitored photometrically using a T<>T containing oligo(dT)_18_. To this end, CPDs and T(6-4)T photoproducts were created by illuminating the oligo(dT)_18_ for 4 h with UV-C on ice (UV-C: GE Healthcare, G15T87B, 15 W; 10 cm distance). A CPD does essentially not absorb at 265 nm while the repaired thymines do. Thus, a repair of CPDs is indicated by an absorbance increase at 265 nm [64]. In contrast, T(6-4)T photoproducts absorb at 325 nm and the decrease in absorbance at 325 nm corresponds to the repair of (6-4) photoproducts [65].

## Supporting Information

Figure S1 **Sequence alignment of the** ***Agrobacterium tumefaciens*** **cryptochrome/photolyase member proteins (AgrtuPhrA and AgrtuPhrB) with representatives of all major classes of the CPF.** Highlighted in red are the conserved Trp residues of the electron-transfer chain (W382–W359–W306 in EsccoPhr1) and those of a proposed alternative electron-transfer chain for CPD II photolyases (W394–W387–W366 in ArathPlr2; modified from [20]). The conserved Trp and Tyr (Y) residues of the FeS-BCPs are highlighted in light blue, the conserved Cys residues, coordinating most likely the (4Fe-4S) cluster, in green. Abbreviations: A. thaliana (Arath), *A. tumefaciens* (Agrtu) *Caulobacter crescentus* (Caucr), *D. melanogaster* (Drome), *E. coli* (Escco), *H. sapiens* (Homsa), *Oceanocaulix alexandrii* (Oceal), *O. sativa* (Orysa), *Rhodobacter sphaeroides 2.4.1* (Rhosp), *Sphingomonas sp.* SKA58 (Sphsp), *Synechocystis sp.* PCC6803 (S6803) and *V. cholerae* (Vibch)). Sequence alignment was generated using ClustalW2.
(DOC)

Table S1 **Sequencing and PCR primers.**
(DOC)
