# Supplementary material for: A Photolyase-Like Protein from Agrobacterium tumefaciens with an Iron-Sulfur Cluster
Source: PLoS One. 2011 Oct 31;6(10):e26775. doi: 10.1371/journal.pone.0026775 (PMC3204975; doi:10.1371/journal.pone.0026775)
Supplement: Figure S1 — Sequence alignment of the Agrobacterium tumefaciens cryptochrome/photolyase member proteins (AgrtuPhrA and AgrtuPhrB) with representatives of all major classes of the CPF. Highlighted in red are the conserved Trp residues of the electron-transfer chain (W382–W359–W306 in EsccoPhr1) and those of a proposed alternative electron-transfer chain for CPD II photolyases (W394–W387–W366 in ArathPlr2; modified from [20]). The conserved Trp and Tyr (Y) residues of the FeS-BCPs are highlighted in light blue, the conserved Cys residues, coordinating most likely the (4Fe-4S) cluster, in green. Abbreviations: A. thaliana (Arath), A. tumefaciens (Agrtu) Caulobacter crescentus (Caucr), D. melanogaster (Drome), E. coli (Escco), H. sapiens (Homsa), Oceanocaulix alexandrii (Oceal), O. sativa (Orysa), Rhodobacter sphaeroides 2.4.1 (Rhosp), Sphingomonas sp. SKA58 (Sphsp), Synechocystis sp. PCC6803 (S6803) and V. cholerae (Vibch)). Sequence alignment was generated using ClustalW2. (DOC) [file pone.0026775.s001.doc]

Fig. S1

*Agrtu* PhrA E-KSAG--------PLGGAQEWWLHHSLAALSSSLEKAG---GRLVLA-SGDAERILRDL 87 CPD III

*Caucr* Phr D-ETPGI------RPMGGASLWWLDKSLKSLAASLETLG---TKLVLR-KGVAAEVLDQL 98

*Arath* Cry1 APEEEGH------YHPGRVSRWWLKNSLAQLDSSLRSLG---TCLITKRSTDSVASLLDV 96 plant Cry

*Orisa* Cry1a APEEDGP------YYPGRVSRWWLSQSLKHLDASLRRLGA--SRLVTRRSADAVVALIEL 104

*Escco* Phr1 TPRQWAT------HNMSPRQAELINAQLNGLQIALAEKGIPLLFREVDDFVASVEIVKQV 91 CPD I

*Vibch* Phr TPEQWHQ------HHLAPIQADLIWRRLAELQQELAALNVPLFYQQVADFQAAAVAVSQL 88

*Arath* CDASH DPRLFHTTHFFNFPKTGALRGGFLMECLVDLRKNLMKRG----LNLLIRSGKPEEILPSL 131 DASH

*S6803* DASH DPRQFAQTHQG-FAKTGPWRSNFLQQSVQNLAESLQKVG----NKLLVTTGLPEQVIPQI 95

*Drome* PL64 DPGILDW------MQVGANRWRFLQQTLEDLDNQLRKLN----SRLFVVRGKPAEVFPRI 96 (6-4) and

*Homsa* Cry1 DPWFAGS------SNVGINRWRFLLQCLEDLDANLRKLN----SRLFVIRGQPADVFPRL 87 animal CRY

*Drome* Cry DGESAGT------KNVGYNRMRFLLDSLQDIDDQLQAATDG-RGRLLVFEGEPAYIFRRL 95

*Arath* Plr2 FDQFLDA---------KARQLGFMLKGLRQLHHQIDSLQ----IPFFLLQGDAKETIPNF 113 CPD II

*Drome* Phr VPKFLNA---------TIRHYKFMMGGLQEVEQQCRALD----IPFHLLMGSAVEKLPQF 285

*Agrtu* PhrB EASYVGH---------HKKKIAFIFSAMRHFAEELRGEGYRVRYTRIDDADNAGSFTGEV 87 FeS-BCP

*Sphsp* Plr ETAYVKH---------HKAKIAFILSAMRHHAERLRALGWTVDYVTLDAPDNRGSFTSEV 90

*Oceal* Plr EVSYVPH---------HRKKIAFLFSAMRHFASRLEGGGKSVRYVRLDDPDNTGSLKGEM 86

*Rhosp* CryB EGTYVPH---------HPQKIALILAAMRKFARRLQERGFRVAYSRLDDPDTGPSIGAEL 87

*Agrtu* PhrA ISETGADTVVWNRRYDPTGMATDKALKQKLRDDG--LTVRSFSGQLLHEPSRLQTKSGG- 144

*Caucr* Phr IAQSGARSVVWNRLYDKPSTDRDAAIKAALRDRG--VDCQSFNAGLLNEPWTVKNGSDQ- 155

*Arath* Cry1 VKSTGASQIFFNHLYDPLSLVRDHRAKDVLTAQG--IAVRSFNADLLYEPWEVTDELGR- 153

*Orisa* Cry1a VRSIGATHLFFNHLYDPLSLVRDHRVKALLTAEG--IAVQSFNADLLYEPWEVVDDDGC- 161

*Escco* Phr1 CAENSVTHLFYNYQYEVNERARDVEVERALRN----VVCEGFDDSVILPPGAVMTGNHE- 146

*Vibch* Phr AKTLNATQVLANRDYELDEQQRDQLAQQLLSEQG--IIWSAFDDKCVLPPGSVRTKQGE- 145

*Arath* CDASH AKDFGARTVFAHKETCSEEVDVERLVNQGLKRVGNSTKLELIWGSTMYHKDDLPFDVFD- 190

*S6803* DASH AKQINAKTIYYHREVTQEELDVERNLVKQLTILG--IEAKGYWGSTLCHPEDLPFSIQD- 152

*Drome* PL64 FKSWRVEMLTFETDIEPYSVTRDAAVQKLAKAEG--VRVETHCSHTIYNPELVKAKNLGK 154

*Homsa* Cry1 FKEWNITKLSIEYDSEPFGKERDAAIKKLATEAG--VEVIVRISHTLYDLDKIIELNGGQ 145

*Drome* Cry HEQVRLHRICIEQDCEPIWNERDESIRSLCRELN--IDFVEKVSHTLWDPQLVIETNGGI 153

*Arath* Plr2 LTECGASHLVTDFSPLREIRRCKDEVVKRTSDSLAIHEVDAHNVVPMWAASSKLEYSAR- 172

*Drome* Phr VKSKDIGAVVCDFAPLRLPRQWVEDVGKALPKSVPLVQVDAHNVVPLWVASDKQEYAAR- 344

*Agrtu* PhrB KRAIDDLTPSRICVTEPGEWRVRSEMDGFAGAFGIQVDIRSDRRFLSSHGEFRNWAAGRK 147

*Sphsp* Plr ARAVERHQPRAIHVTEAGEWRVRSMIESWETRFAIPVTIHEDDRFLCSHAEFDSWAAARN 150

*Oceal* Plr ERALKAGAFDEVVVVEPGEYRLKAEFEQWADTFDVPVRMRRDDRFICTLDRFNAWAEGRK 146

*Rhosp* CryB LRRAAETGAREAVATRPGDWRLIEALEAMP----LPVRFLPDDRFLCPADEFARWTEGRK 143

*Agrtu* PhrA ---PYRVYTPFWRALEGSDEPH---------APADPPKSLTAPKVWPKSEKLSNWKLLP- 191

*Caucr* Phr ---PYKVFTPYWRAAREHLTDV---------AVTAAPGHLVAPARFPASESLASWNLHP- 202

*Arath* Cry1 ---PFSMFAAFWERCLSMPYDP---------ESPLLPPKKIISGDVS--KCVADPLVFED 199

*Orisa* Cry1a ---PFTMFAPFWDRCLCMP-DP---------AAPLLPPKRIAPGELPARRCPSDELVFED 208

*Escco* Phr1 ---MYKVFTPFKNAWLKRLR------------EGMPECVAAPKVRSS-GSIEPSPSITLN 190

*Vibch* Phr ---FFKVFTPFKRAWLTLFQPP---------VIGKNRPVALWNVPSALAELVWHPEQAFD 193

*Arath* CDASH ---LPDVYTQFRKSVEAK-CSI---------RSSTRIPLSLGPTPSVDDWGDVPTLEKLG 237

*S6803* DASH ---LPDLFTKFRKDIEKKKISI---------RPCFFAPSQLLPSPNIKLELTAPPPEFFP 200

*Drome* PL64 APITYQKFLGIV---EQLKVPK---------VLGVPEKLKKMPTPPKD-EVEQKDSAAYD 201

*Homsa* Cry1 PPLTYKRFQTLISKMEPLEIPV---------ETITSEVIEKCTTPLSD-DHDEK----YG 191

*Drome* Cry PPLTYQMFLHTV---QIIGLPP---------RPTADARLEDATFVELDPEFCRSLKLFEQ 201

*Arath* Plr2 --TIRGKINKLLPDYLIEFPKL---------EPPKKKWTGMMDKKLVDWDSLIDKVVREG 221

*Drome* Phr --TIRNKINSKLGEYLSEFPPV---------VR-HPHGTGCKNVNTVDWSAAYASLQCD- 391

*Agrtu* PhrB SLTMEYFYREMRRKTGLLMNGE-QPVGGRWNFDAENRQPAR-PDLLRPKHPVFAPDKITK 205

*Sphsp* Plr QLRMEFFYRDMRRKTGLLMTDAGEPEGGQWNYDADNRKPAPDRDLLMPHPIRFRPDGVTQ 210

*Oceal* Plr RLTMEYFYREMRRETGLLMDGD-EPEGGQWNFDKDNRKALP-DELTPPERAFIEPDALTR 204

*Rhosp* CryB QLRMEWFYREMRRRTGLLMEGD-EPAGGKWNFDTENRKPAA-PDLLRPRPLRFEPDAEVR 201

*Agrtu* PhrA -TKPDWAKDFSD--------IWTPGETGALDKLDDFIDGALKGYE-----EGRD-FPAKP 236

*Caucr* Phr -TKPDWSKGF-D--------LWTPGEAGAHARLDAFLKGPIKGYG-----DQRD-IPGVE 246

*Arath* Cry1 DSEKGSNALLAR--------AWSPGWSNGDKALTTFINGPLLEYS-----KNRR-KADSA 245

*Orisa* Cry1a ESERGSNALLAR--------AWSPGWQNADKALAAFLNGPLMDYS-----VNRK-KADSA 254

*Escco* Phr1 YPRQSFDTAH-----------FPVEEKAAIAQLRQFCQNGAGEYE-----QQRD-FPAVE 233

*Vibch* Phr YPR--IDSTP-----------WAADFETVRAQLRDFCRERVQDYH-----QARD-FPARE 234

*Arath* CDASH VEPQEVTRGMRF--------VGGESAGVGRVFEYFWKKDLLKVYK-----ETRNGMLGPD 284

*S6803* DASH QINFDHRSVLAF--------QGGETAGLARLQDYFWHGDRLKDYK-----ETRNGMVGAD 247

*Drome* PL64 CPTIKQLVKRPE---ELGPNKFPGGETEALRRMEESLKDEIWVAR--FEKPNTAPNSLEP 256

*Homsa* Cry1 VPSLEELGFDTD---GLSSAVWPGGETEALTRLERHLERKAWVAN--FERPRMNANSLLA 246

*Drome* Cry LPTPEHFNVYGDNMGFLAKINWRGGETQALLLLDERLKVEQHAFERGFYLPNQALPNIHD 261

*Arath* Plr2 AEVPEIEWCVPG---------EDAGIEVLMGNKDGFLTKRLKNYS-----TDRNNPIKPK 267

*Drome* Phr MEVDEVQWAKPG---------YKAACQQLY----EFCSRRLRHFN-----DKRNDPT-AD 432

*Agrtu* PhrB EVIDTVERLFPDNFGKLENFGFAVTRTDAERALSAFIDDFLCNFG--ATQDAMLQDDPNL 263

*Sphsp* Plr AVLDMVADRFADHIGSLDYFHFAVTHDEALRQRKRFLDDALPRFG--DYQDAMLTDEPFL 268

*Oceal* Plr EVLALVEARFPDQFGDLEAFGYAVTQEDAAAQLDWFIENGLPCFG--DYQDALKQEEAFL 262

*Rhosp* CryB AVLDLVEARFPRHFGRLRPFHWATDRAEALRALDHFIRESLPRFG--DEQDAMLADDPFL 259

*Agrtu* PhrA ATSLLSPHLAAGEISPAAVWHATKG-----LSR--HIASN--DISRFRKEIVWREFCYHL 287 CPD III

*Caucr* Phr ATSKLSPHLHFGEIGPRQVWLATRS-----AADQGDIPLA--EADKFLSEIGWREFNHSI 299

*Arath* Cry1 TTSFLSPHLHFGEVSVRKVFHLVRIKQVAWANEGNEAGEE--SVNLFLKSIGLREYSRYI 303 plant Cry

*Orisa* Cry1a STSLLSPYLHFGELSVRKVFHQVRMKQLMWSNEGNHAGDE--SCVLFLRSIGLREYSRYL 312

*Escco* Phr1 GTSRLSASLATGGLSPRQCLHRLLAE------QPQALDGG--AGSVWLNELIWREFYRHL 285 CPD I

*Vibch* Phr GTSSLSPYLAIGVLSARQCVARLYHE------SSMGELSE--GAQVWLSELIWREFYQHL 286

*Arath* CDASH YSTKFSPWLAFGCISPRFIYEEVQRY------EKERVANN--STYWVLFELIWRDYFRFL 336 DASH

*S6803* DASH YSSKFSPWLALGCLSPRFIYQEVKRY------EQERVSND--STHWLIFELLWRDFFRFV 299

*Drome* PL64 STTVLSPYLKFGCLSARLFNQKLKEIIKRQPKHSQPP-------VSLIGQLMWREFYYTV 309 (6-4) and

*Homsa* Cry1 SPTGLSPYLRFGCLSCRLFYFKLTDLYKKVKKNSSPP-------LSLYGQLLWREFFYTA 299 animal CRY

*Drome* Cry SPKSMSAHLRFGCLSVRRFYWSVHDLFKNVQLRACVRGVQMTGGAHITGQLIWREYFYTM 321

*Arath* Plr2 ALSGLSPYLHFGQVSAQRCALEARKVR----STSPQAVDIFLEELIVRRELSDNFCYYQP 323 CPD II

*Drome* Phr ALSGLSPWLHFGHISAQRCALEVQRFR----GQHKASADAFCEEAIVRRELADNFCFYNE 488

*Agrtu* PhrB NHSLLSFYINCGLLDALDVCKAAERAYH------EGGAPLNAVEGFIRQIIGWREYMRGI 317 FeS-BCP

*Sphsp* Plr WHSILSPYINAGLLDPLDLCREVEARYR------AGKVPLNCAEGFIRQIIGWREYVRGV 322

*Oceal* Plr FHSVLSLYLNCGLLDPLEVCRRAEAAYK------QGQAPLNAVEGFIRQILGWREYVRGV 316

*Rhosp* CryB SHALLSSSMNLGLLGPMEVCRRAETEWR------EGRAPLNAVEGFIRQILGWREYVRGI 313

*Agrtu* PhrA LFHFPELGE-KNWNDSFDAFSWRD-DEKSFKAWTRGMTGYPIVDAGMRQLWQHGTMHNRV 345

*Caucr* Phr LYNWPHMPS-ANFKPEFDGFPWVK-DEGALEAWKRGQTGYPIVDAGMRELWTTGFMHNRV 357

*Arath* Cry1 SFNHPYSHE-RPLLGHLKFFPWAV-DENYFKAWRQGRTGYPLVDAGMRELWATGWLHDRI 361

*Orisa* Cry1a TFNHPCSLE-KPLLAHLRFFPWVV-DEVYFKVWRQGRTGYPLVDAGMRELWATGWLHDRI 370

*Escco* Phr1 ITYHPSLCKHRPFIAWTDRVQWQS-NPAHLQAWQEGKTGYPIVDAAMRQLNSTGWMHNRL 344

*Vibch* Phr VAIEPNLSKSRDFVEWGARLEWWN-DNEKFQLWCEGKTGYPIVDAAMRQLNQTGWMHNRL 345

*Arath* CDASH SIKCGNSLFHLGGPRNVQ-GKWSQ-DQKLFESWRDAKTGYPLIDANMKELSTTGFMSNRG 394

*S6803* DASH AQKYGNKLFNRGGLLNKN-FPWQE-DQVRFELWRSGQTGYPLVDANMRELNLTGFMSNRG 357

*Drome* PL64 AAAEPNFDR-MLGNVYCMQIPWQE-HPDHLEAWTHGRTGYPFIDAIMRQLRQEGWIHHLA 367

*Homsa* Cry1 ATNNPRFDK-MEGNPICVQIPWDK-NPEALAKWAEGRTGFPWIDAIMTQLRQEGWIHHLA 357

*Drome* Cry SVNNPNYDR-MEGNDICLSIPWAKPNENLLQSWRLGQTGFPLIDGAMRQLLAEGWLHHTL 380

*Arath* Plr2 HYDSLKGAWEWARKSLMDHASDKREHIYSLEQLEKGLTADPLWNASQLEMLYQGKMHGFM 383

*Drome* Phr HYDSLKGLSSWAYQTLDAHRKDKRDPCYSLEELEKSLTYDDLWNSAQLQLVREGKMHGFL 548

*Agrtu* PhrB YWLAG---------PDYVDSNFFENDRSLPVFYWTGKTHMNCMAKVITETIENAYAHHIQ 368

*Sphsp* Plr YWHEG---------PDYGKRNALEAQRDLPDFYWTGETDMHCLAQAIGQTIDHGYAHHIQ 373

*Oceal* Plr YWRFM---------PDYLERNALEAKRNLPDFYWSADTDMACVRDVIITTRKHAYAHHIQ 367

*Rhosp* CryB WTLSG---------PDYIRSNGLGHSAALPPLYWGKPTRMACLSAAVAQTRDLAYAHHIQ 364

*Agrtu* PhrA RMIVASFLIK-HLLIDWRKGEKWFRD--TLVDADPASNAANWQWVAGSGADASPFFR-IF 401

*Caucr* Phr RMIVASFLIK-HLMIDWREGEAWFWD--TLLDADLANNVGNWQWTAGSGADAAPYFR-IF 413

*Arath* Cry1 RVVVSSFFVK-VLQLPWRWGMKYFWD--TLLDADLESDALGWQYITGTLPDSREFDR-ID 417

*Orisa* Cry1a RVVVSSFFVK-VLQLPWRWGMKYFWD--TLLDADLESDALGWQYISGSLPDGRELDR-ID 426

*Escco* Phr1 RMITASFLVK-DLLIDWREGERYFMS--QLIDGDLAANNGGWQWAASTGTDAAPYFR-IF 400

*Vibch* Phr RMIVASFLTK-DLHIDWRWGERYFMS--RLIDGDYAANNGGWQWCASTGCDGQPYFR-IF 401

*Arath* CDASH RQIVCSFLVR-DMGLDWRMGAEWFET--CLLDYDPCSNYGNWTYGAGVGNDPR-EDR-YF 449

*S6803* DASH RQNVASFLCK-NLGIDWRWGAEWFES--CLIDYDVCSNWGNWNYTAGIGNDAR-DFR-YF 412

*Drome* PL64 RHAVACFLTRGDLWISWEEGQRVFEQ--LLLDQDWALNAGNWMWLSASAFFHQYFR--VY 423

*Homsa* Cry1 RHAVACFLTRGDLWISWEEGMKVFEE--LLLDADWSINAGSWMWLSCSSFFQQFFH--CY 413

*Drome* Cry RNTVATFLTRGGLWQSWEHGLQHFLK--YLLDADWSVCAGNWMWVSSSAFERLLDSSLVT 438

*Arath* Plr2 RMYWAKKILEWTKGPEEALSISIYLNNKYEIDGRDPSGYVGCMWSICGVHDQGWKERPVF 443

*Drome* Phr RMYWAKKILEWTATPEHALEYAILLNDKYSLDGRDPNGYVGCMWSIGGVHDMGWKERAIF 608

*Agrtu* PhrB RLMITGNFALLAGIDPKAVHRWYLEVYADAYEWVELPNVIGMSQFADGGFLGTKPYAASG 428

*Sphsp* Plr RLMITGNFALIAGIDPHQVHVWYLEVYADAYEWVEMPNTIGMALFADGGLLGSKPYAAGG 433

*Oceal* Plr RLMVTGNFALLAGVDPKAVNEWYLAVYADAYEWVEAPNTHGMALFADGGLMGTKPYAASG 427

*Rhosp* CryB RLMVTGNFALLAGVDPAEVHEWYLSVYIDALEWVEAPNTIGMSQFADHGLLGSKPYVSSG 424

*Agrtu* PhrA NPILQGEKFDGDGDYVRRFVPELEKLERKYIHKP---FEAPKDALKKAGVELGKTYPLPI 458

*Caucr* Phr NPIAQGEKFDPKGDYVRRWVPELRNVSDDVIHKP---WTKPLHLPAGA----KRLYSRPI 466

*Arath* Cry1 NPQFEGYKFDPNGEYVRRWLPELSRLPTDWIHHP---WNAPESVLQAAGIELGSNYPLPI 474

*Orisa* Cry1a NPQLEGYKFDPHGEYVRRWLPELARLPTEWIHHP---WDAPESVLQAAGIELGSNYPLPI 483

*Escco* Phr1 NPTTQGEKFDHEGEFIRQWLPELRDVPGKVVHEP---WKW------AQKAGVTLDYPQPI 451

*Vibch* Phr NPVSQGEKFDPNGDFIRRWVPELRSVSSAYIHQP---WTY------PAVN--SVLYPARL 450

*Arath* CDASH SIPKQAQNYDPEGEYVAFWLQQLRRLPKEKRHWPGRLMYMDTVVPLKHGNGPMAGGSKSG 509

*S6803* DASH NIPKQSQQYDPQGTYLRHWLPELKNLPGDKIHQP---WLLSATEQKQWGVQLGVDYPRPC 469

*Drome* PL64 SPVAFGKKTDPQGHYIRKYVPELSKYPATCIYEP---WKASLVDQRAYGCVLGTDYPHRI 480

*Homsa* Cry1 CPVGFGRRTDPNGDYIRRYLPVLRGFPAKYIYDP---WNAPEGIQKVAKCLIGVNYPKPM 470

*Drome* Cry CPVALAKRLDPDGTYIKQYVPELMNVPKEFVHEP---WRMSAEQQEQYECLIGVHYPERI 495

*Arath* Plr2 GKIRYMNYAGCKRKFNVDSYISYVKSLVSVTKKK-----------RKAEEQLTRDSVDPK 492

*Drome* Phr GKVRYMNYQGCRRKFDVNAFVMRYG--GKVHKKK-------------------------- 640

*Agrtu* PhrB NYINRMSDYCDTCRYDPKERLGDNACPFNALYWD---FLARNREKLKSNHRLAQPYATWA 485

*Sphsp* Plr AYINRMSNYCGTCRYDVKKRVGDDACPFNALYWD---FIARNERRLARNPRMAMPYRNWQ 490

*Oceal* Plr SYINKMSDHCKQCSYAVSKKNGPKACPFNYLYWN---FLMENRDRLEGNHRLSMIYKTLD 484

*Rhosp* CryB AYIDRMSDYCRGCAYAVKDRTGPRACPFNLLYWH---FLNRHRARFERNPRMVQMYRTWD 481
