# Supplementary material for: A Photolyase-Like Protein from Agrobacterium tumefaciens with an Iron-Sulfur Cluster
Source: PLoS One. 2011 Oct 31;6(10):e26775. doi: 10.1371/journal.pone.0026775 (PMC3204975; doi:10.1371/journal.pone.0026775)
Supplement: Table S1 — Sequencing and PCR primers. (DOC) [file pone.0026775.s002.doc]

Table S1: Sequencing and PCR primers.

| Primer | Sequence |
| --- | --- |
| tpnRL17–1 | AACAAGCCAGGGATGTAACG |
| tpnRL13–2 | CAGCAACACCTTCTTCACGA |
| *phrA* 5` | TCGCTAAAAACCGCCCCC |
| *phrA* 3` | TTATGTGGTCTTCTTCACTGCG |
| *phrB* NdeI 5` | ACTCCATATGTCTCAGCTGGTGTTGATCCT |
| *phrB* NotI 3` | ATAGCGGCCGCGTCAAGCTTTCGCAGAAAA |
| probe 5` | ATAGGACGATGCTGGATGTCGAGGTGTCGTTAATGTG |
| probe 3` | CCGTATAGTACGACCTACAGCTCCACATTAACGAC |
